# Assessing knowledge and awareness of dental practitioners in detecting, evaluating, and managing pre-eruptive intracoronal resorption: a cross-sectional study

**DOI:** 10.3389/froh.2026.1880719

**Published:** 2026-09-30

**Authors:** Amani A. Al Tuwirqi, Jamila A. Almashaikhi, Osama M. Felemban, Heba M. Elkhodary

**Affiliations:** 1 Pediatric Dentistry Department, Faculty of Dentistry, King Abdulaziz University, Jeddah, Saudi Arabia; 2 Department of Pedodontics and Oral Health, Faculty of Dental Medicine for Girls, Alazhar University, Cairo, Egypt

**Keywords:** awareness, dental practitioners, diagnosis, knowledge, management, pre-eruptive intracoronal resorption, radiographs

## Abstract

This study aimed to evaluate the proficiency of dental practitioners at King Abdulaziz University Dental Hospital (KAUDH) in detecting and diagnosing pre-eruptive intracoronal resorption (PEIR). It also sought to examine their knowledge of PEIR etiology, severity, and management, while investigating how factors such as educational level and clinical experience influence their detection capabilities and overall understanding of the condition.

**Materials and methods:**

This cross-sectional study was conducted at KAUDH and involved 125 participants. The participants were categorized into four subgroups based on their educational level: interns, general dentists, postgraduate pediatric dentistry residents, and postgraduate residents from other specialties. Each participant was asked to examine eight orthopantomograms, which included one normal radiograph, one case of tooth impaction, one case of odontodysplasia, and five cases of PEIR with varying severities. Participants were also required to answer four general questions about PEIR using a validated questionnaire.

**Results:**

The results showed that the participants were generally proficient in the radiographic diagnosis and interpretation of common and obvious anomalies. However, they had difficulty recognizing and identifying PEIR lesions. As the severity of PEIR increased, participants were better able to identify the affected teeth; however, awareness of the correct management decreased. Pediatric dentistry residents and participants with ≥5 years of clinical experience demonstrated better diagnostic ability, both generally and specifically in relation to PEIR, than other dental practitioners. The participants had limited knowledge of PEIR. Only 24.8% of the participants had heard of PEIR, and only 6.4% had encountered a PEIR lesion in their clinics. Furthermore, only 8.8% of the participants knew that PEIR lesions are caused by resorption.

**Conclusions:**

The study identified important gaps in awareness and conceptual understanding of PEIR among dental practitioners, particularly regarding its etiology and management. Recognition of subtle PEIR lesions was limited, although participants demonstrated improved detection as lesion severity increased. Diagnostic accuracy and radiographic interpretation were positively associated with higher educational levels and greater clinical experience. These findings highlight the need for enhanced undergraduate and continuing dental education to improve the early detection and appropriate management of PEIR lesions.

## 1 Introduction

Pre-eruptive intracoronal resorption (PEIR) is a condition affecting the coronal dentin of unerupted primary and permanent teeth (1). These lesions are typically discovered incidentally during routine radiographic examinations and manifest as well-circumscribed radiolucent areas adjacent to the dentinoenamel junction (DEJ) (2). PEIR lesions primarily occur in the central region of the coronal dentin because of disruption of the protective enamel epithelium and subsequent invasion by resorptive cells (2, 3). Histological analyses have provided evidence of the resorptive nature of PEIR, revealing scalloped margins and the presence of chronic inflammatory cells, osteoclasts, macrophages, and multinucleated giant cells originating from the surrounding bone (1, 4, 5).

The prevalence of PEIR in the permanent dentition ranges from 0.6% to 27.3% (3, 6–11). Although its prevalence in primary teeth remains unknown because radiographs are infrequently obtained for unerupted primary teeth (6), early diagnosis is crucial for improving outcomes, minimizing the risk of pulpal involvement, and preventing tooth extraction (2, 8). Various classification systems, including Seow's classification and its subsequent modifications, categorize PEIR lesions according to their size and extent of involvement (8, 10, 12, 13).

Management strategies for PEIR depend on factors such as lesion size, expected eruption time, progression rate, and individual patient considerations. PEIR lesions may be classified as either non-progressive or progressive. The rate of progression can vary, even among individuals with multiple affected teeth, necessitating periodic radiographic examinations every 6–12 months to monitor lesion progression (2, 14). According to these factors, different treatment options for PEIR have been reported in the literature. Conservative management is often preferred for PEIR 1 defects or teeth nearing eruption and involves careful monitoring until the tooth erupts and restorative procedures can be safely performed (2, 4, 5, 15). For symptomatic or rapidly progressing lesions, or for teeth with PEIR 2–4 lesions that are not expected to erupt soon, surgical intervention may be necessary. This approach includes surgical exposure of the lesion and curettage to remove soft tissue without the use of a handpiece, thereby minimizing the risk of pulp exposure (2, 4). In cases of pulp exposure, direct pulp capping with materials such as mineral trioxide aggregate or Biodentine is recommended because of their favorable properties compared with traditional calcium hydroxide. A subsequent coronal seal is essential to prevent contamination and deterioration of dental tissues following tooth eruption (2). Finally, in extensive cases in which lesions are unmanageable, extraction may be considered the only viable option for maintaining overall dental health (2, 5). Despite the growing body of literature on PEIR, most studies have been limited to case reports or prevalence studies (6, 10, 15). Notably, only one published study has assessed dental practitioners' awareness of PEIR in South India and found that 61% of practitioners were familiar with the condition (16). Research on PEIR in Saudi Arabia is limited, with only one study reporting a prevalence of 0.6% among Saudi children (3). Therefore, this study aimed to evaluate the knowledge of PEIR among dental practitioners at King Abdulaziz University Dental Hospital (KAUDH). The main objectives were to assess the ability of these practitioners to detect and diagnose PEIR, evaluate their knowledge of PEIR etiology, severity, and management, and investigate how factors such as educational level and clinical experience influence their detection capabilities and overall understanding of the condition.

To align directly with these objectives, the following hypotheses were tested:

- **Hypothesis 1 (Detection & Diagnosis):** Dental practitioners' ability to accurately detect and diagnose radiographs varies significantly when comparing normal versus abnormal radiographs, as well as when identifying obvious abnormalities compared to PEIR.
- **Hypothesis 2 (Educational Level):** Participants' performance on knowledge questions specifically covering PEIR detection, diagnosis, etiology, severity, and management differs significantly based on their educational background (interns, general dentists, pediatric residents, and residents from other specialties).
- **Hypothesis 3 (Clinical Experience):** Participants' performance across the knowledge domains varies significantly according to their years of clinical experience (1 year, 2–4 years, and ≥5 years).

## 2 Materials and methods

### 2.1 Study settings and design

This cross-sectional study was conducted at King Abdulaziz University Dental Hospital, Jeddah, Saudi Arabia (KAUDH) from June to December 2023. The study protocol was approved by the Research Ethics Committee at KAUDH. This study adhered to the guidelines outlined in the Strengthening the Reporting of Observational Studies in Epidemiology statement (STROBE).

### 2.2 Study subjects

All dental interns, general dentists, and postgraduate students enrolled in master's and doctoral programs at KAUDH, Jeddah, Saudi Arabia, were eligible for the study. The present study used the same participant cohort as a previously published study (1); however, the current manuscript reports a different analysis focused on outcomes that were not included in the previous publication, specifically knowledge, recognition, and management of PEIR. Invitation emails were sent by the primary investigator to all eligible candidates. A one-to-one meeting was then arranged with those who responded and agreed to participate, during which written informed consent was obtained. The study participants were divided into four groups: Group 1, dental interns; Group 2, general dentists; Group 3, postgraduate candidates enrolled in pediatric dentistry master's and PhD programs; and Group 4, postgraduate candidates enrolled in other dentistry master's programs.

### 2.3 Sample size

The sample size was calculated based on the assumption of a 1-point difference in the overall questionnaire mean scores of 1 between groups and a common standard deviation of 2. It was determined that a minimum of 21 participants per group would be required to detect statistically significant differences between group means at a significance level of 0.05 with 80% power. The anticipated 1-point difference was considered educationally meaningful among groups with different levels of training and clinical experience because no directly comparable previous studies using the same questionnaire and scoring system were available to inform the effect size estimate.

### 2.4 Questionnaire development and validation

An electronic questionnaire was developed based on a thorough literature review, in addition to the researchers' scientific knowledge and expertise. The questionnaire was validated to ensure clarity, content relevance, and reliability. Five pediatric dentistry trainees first completed the questionnaire and provided feedback on its clarity to establish face validity; these questionnaires were excluded from the final study sample. Four experts in pediatric dentistry then assessed the items for relevance, clarity, simplicity, and ambiguity using a four-point Likert scale through the SMI software (17). The calculated Scale Content Validity Index (S-CVI) was obtained as the average proportion of items rated as content-valid by the experts and was found to be 96.4%, indicating excellent content validity. Minor revisions were subsequently made to five questions based on expert feedback. Formal inter-rater agreement statistics were not calculated. Test–retest reliability was evaluated by asking twenty dentists to complete the questionnaire twice, with a two-week interval between administrations, using a Google Form; these questionnaires were excluded from the final study sample. The Spearman's rho correlation coefficient (*ρ* = 0.927, *p* < 0.001) confirmed the excellent reliability and stability of the instrument.

### 2.5 Study procedures

The questionnaire consisted of three parts. The first part focused on the participants' demographic information. It included six questions related to gender, age, years of clinical experience, specialty, academic level, and country of undergraduate education. The second part consisted of three case-based scenarios. In the first scenario, four orthopantomograms (OPGs) were presented. The first radiograph showed no abnormalities (Figure 1), the second demonstrated an impacted tooth (#35) associated with a dentigerous cyst (Figure 2), the third showed a case of moderate PEIR involving tooth #45 (Figure 3), and the fourth depicted odontodysplasia affecting multiple teeth (#21, #22, #23, and #24) (Figure 4). Two questions followed each radiograph: (Q1) Was there any abnormality? and (Q2) What is/are the abnormality(ies)? The purpose of this scenario was to assess the participants' ability to detect and diagnose normal and abnormal radiographs, as well as to compare their diagnostic accuracy in identifying obvious abnormalities (Case #2, involving an impacted tooth associated with a dentigerous cyst, and Case #4, involving odontodysplasia) with their accuracy in identifying the PEIR case, in which the abnormality was less apparent.

**Figure 1:**
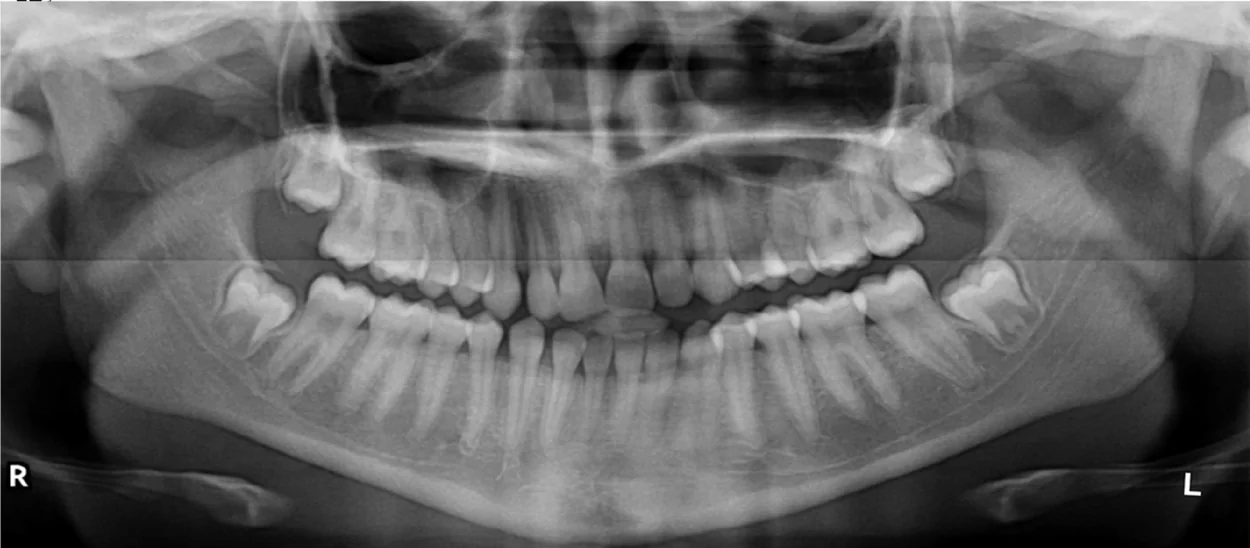
Case 1 - Normal panoramic radiograph without abnormality [18].

**Figure 2:**
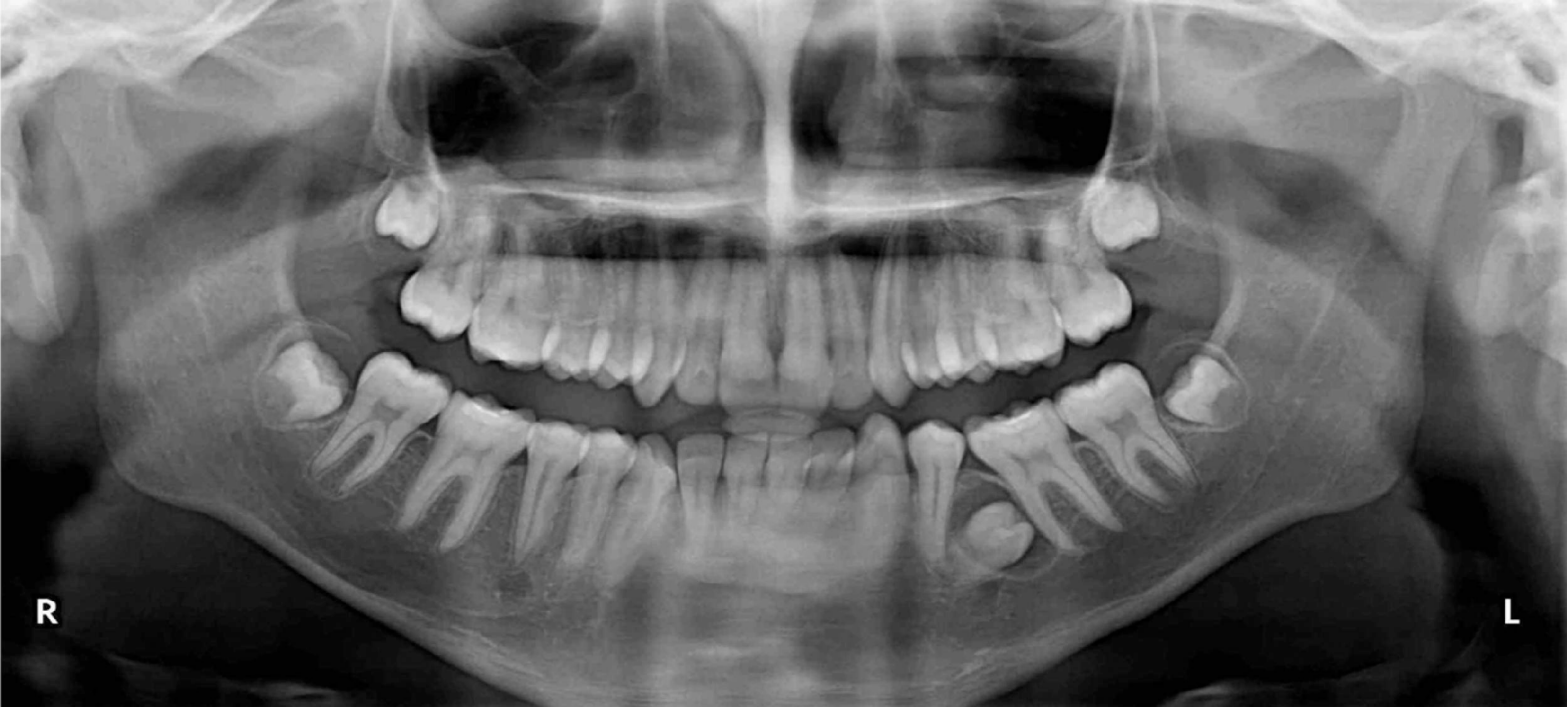
Case 2 - Panoramic radiograph with impacted tooth #35.

**Figure 3:**
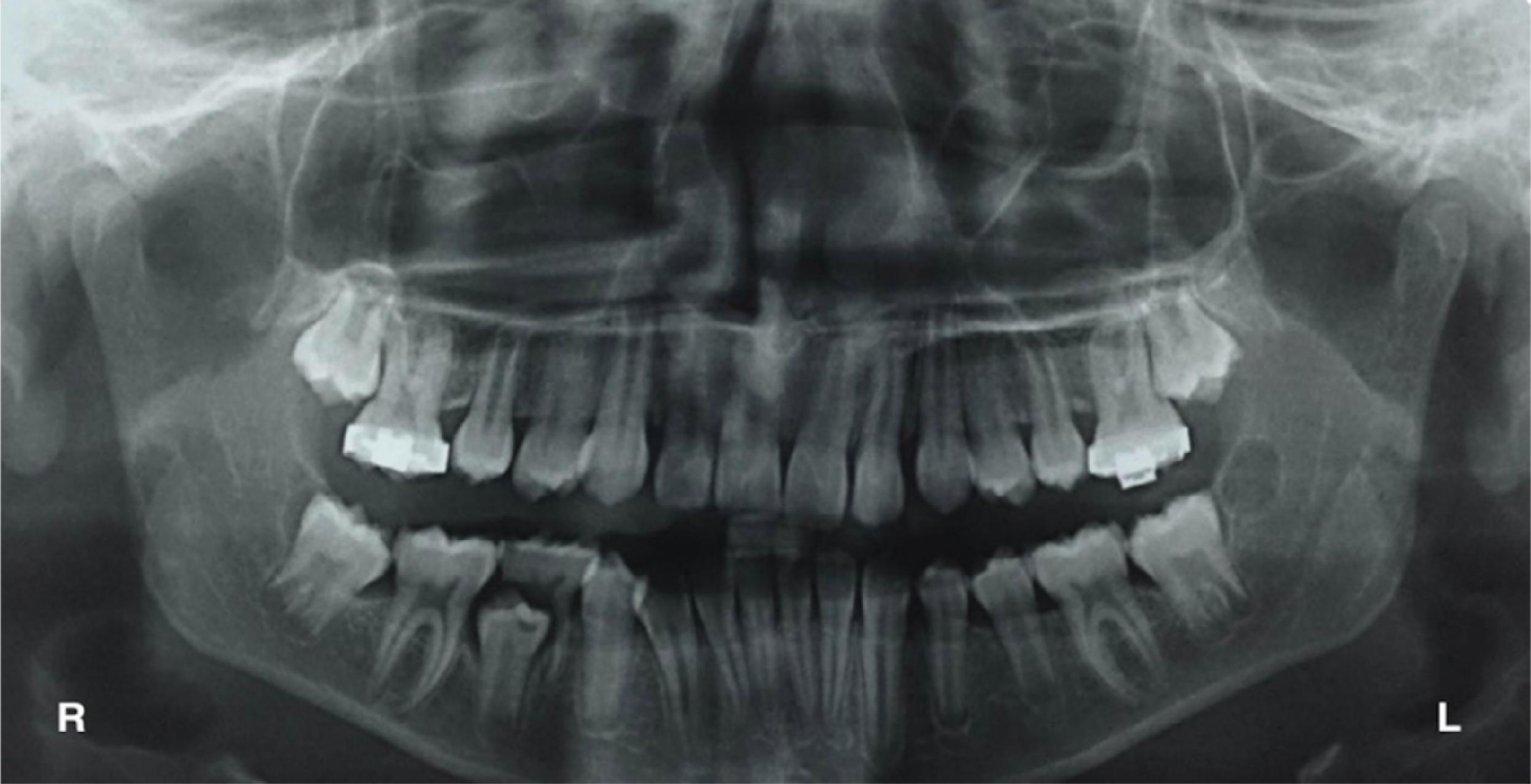
Case 3 - Panoramic radiograph with a moderate size PEIR in tooth #45 [15].

**Figure 4:**
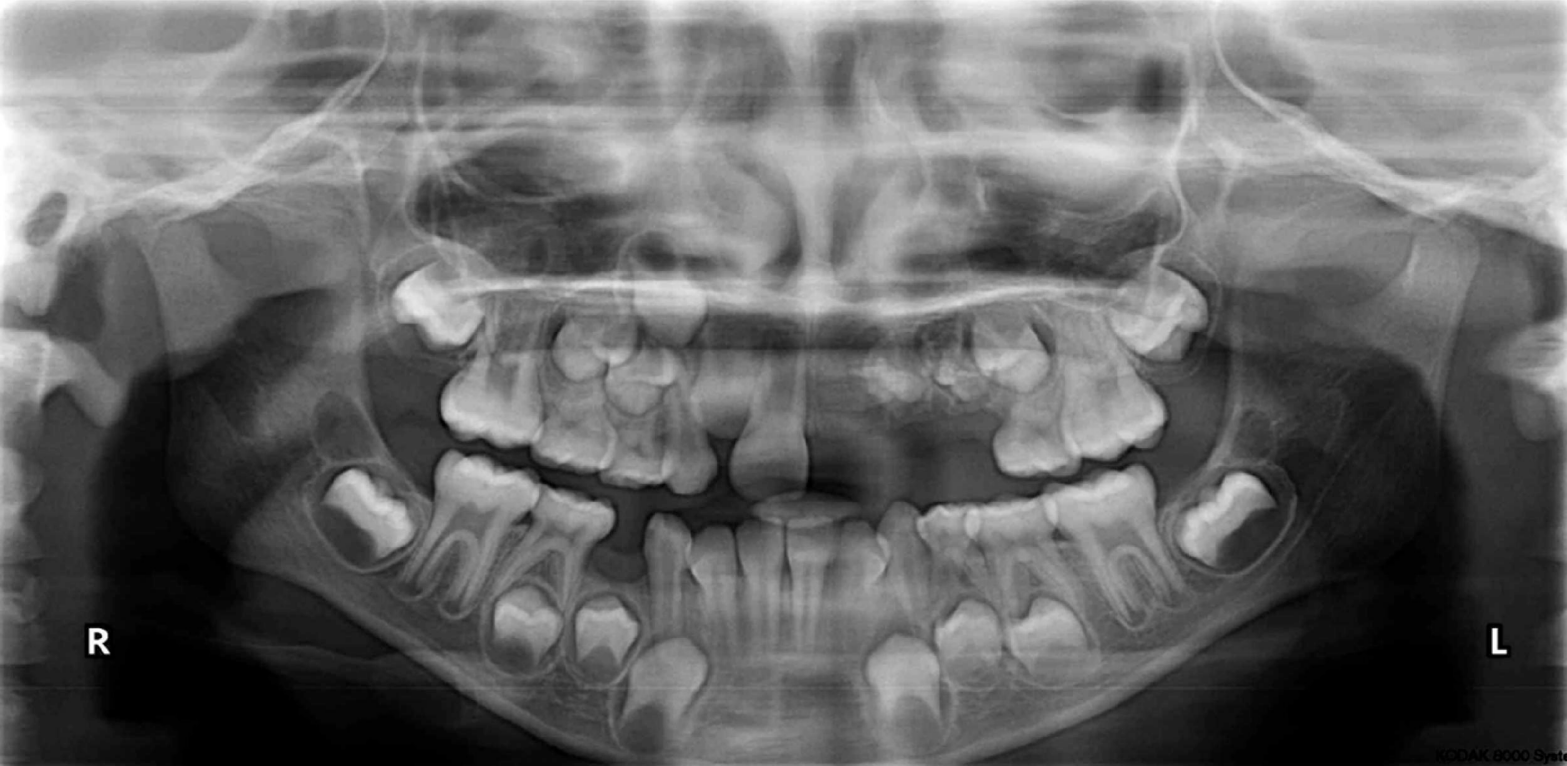
Case 4 - Panoramic radiograph with Odontodysplasia in multiple teeth 21, 22, 23, 24.

The second scenario began by informing the participants that the radiograph contained an abnormality and asking them to identify the affected tooth. This scenario included one OPG in which tooth #38 was affected by moderate PEIR (Figure 5). The case was followed by an open-ended question requiring participants to identify the affected tooth. The purpose of this scenario was to evaluate the effect of prior knowledge that the radiograph contained an abnormality on the participants' ability to detect the tooth affected by PEIR. The third scenario began by informing the participants that the cases presented PEIR lesions. In this scenario, three cases of PEIR were arranged according to lesion severity. The first was a case of mild PEIR affecting tooth #45 (Figure 6), followed by a case of moderate PEIR affecting tooth #36 (Figure 7), whereas the third case showed severe PEIR affecting tooth #47 (Figure 8). Each case was followed by an open-ended question requiring participants to identify the affected tooth and two multiple-choice questions assessing their ability to recognize PEIR severity and select the appropriate management approach. The purpose of this scenario was to assess the ability of dental practitioners to identify teeth affected by PEIR, as well as to evaluate their knowledge of PEIR severity and management.

**Figure 5:**
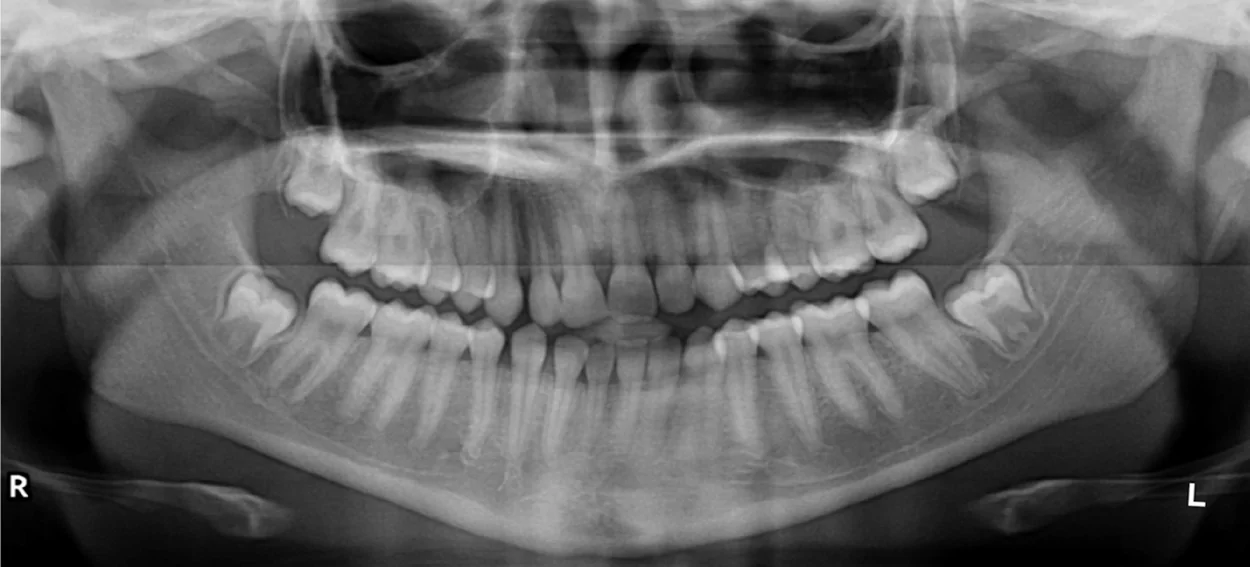
Case 5 - Panoramic radiograph with tooth #38 affected by moderate size PEIR [18].

**Figure 6:**
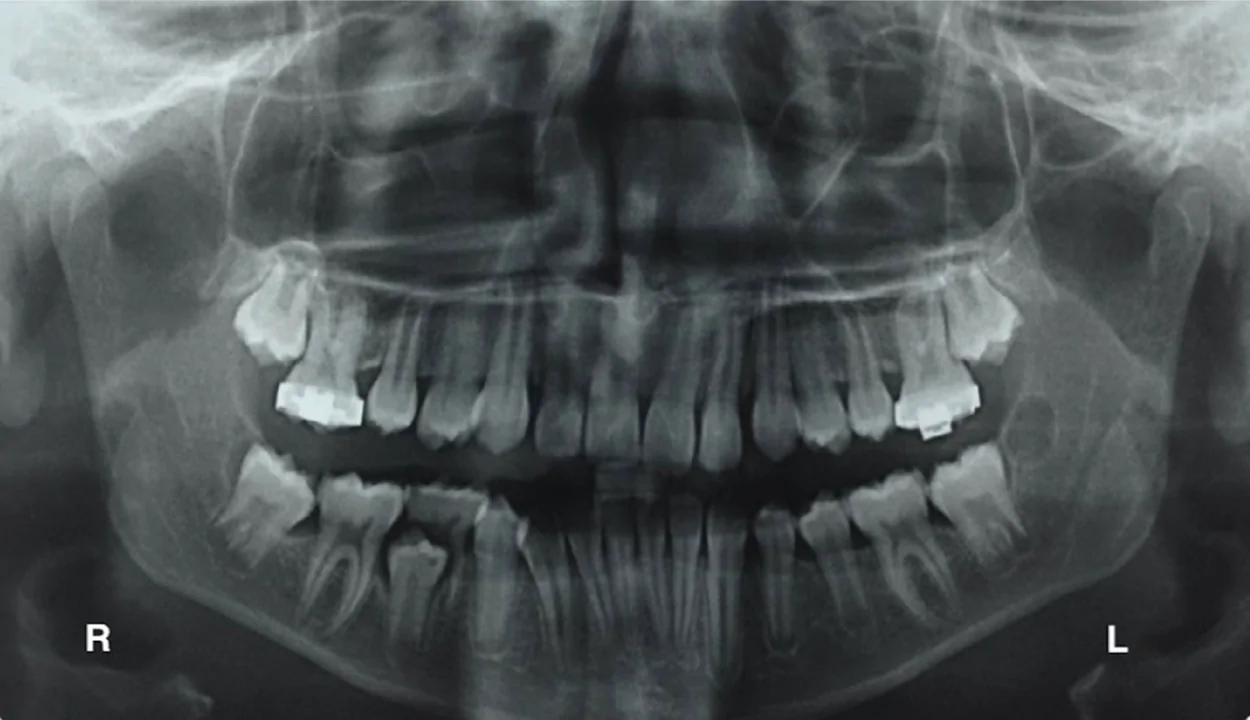
Case 6 - Panoramic radiograph with a mild size PEIR in tooth #45 [15].

**Figure 7:**
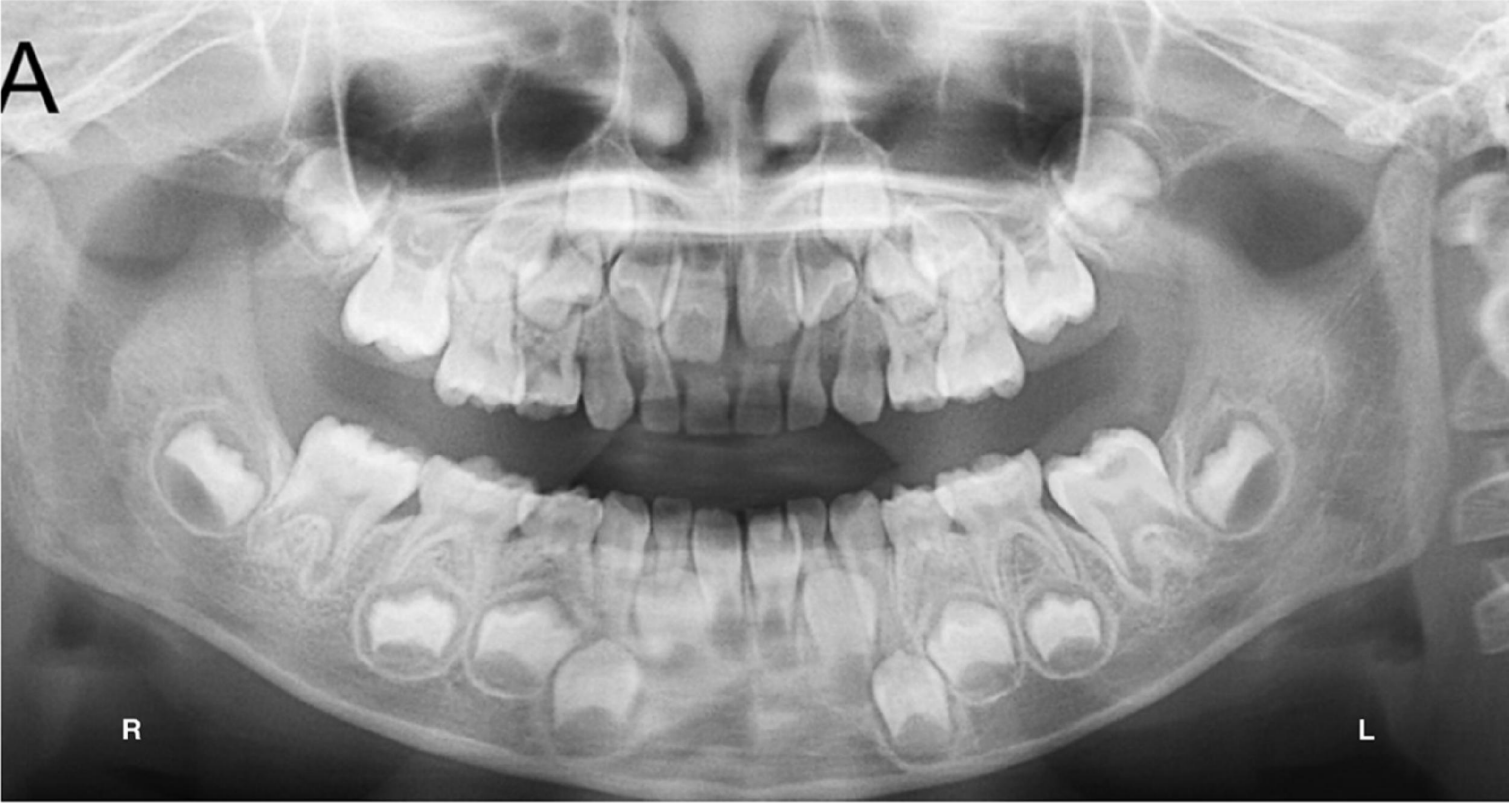
Case 7 - Panoramic radiograph with a moderate size PEIR in tooth #36 [15].

**Figure 8:**
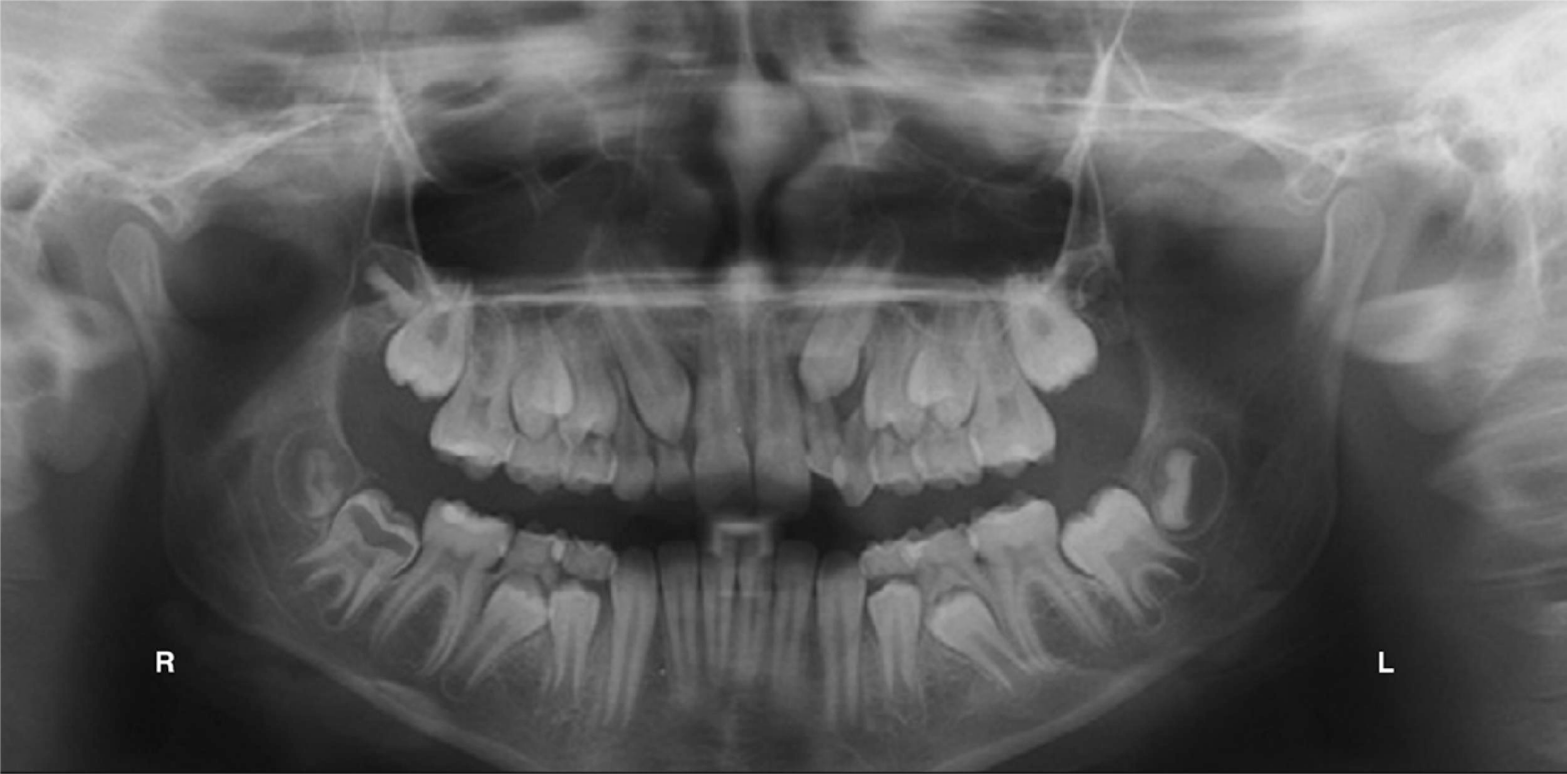
Case 8 - Panoramic radiograph with a severe PEIR in tooth #47 [14].

The third part comprised four questions related to PEIR knowledge and clinical experience. The first question was, “Have you ever heard of PEIR?” with response options of “Yes” or “No.” The second question was, “Have you ever encountered a PEIR lesion in your clinic?” with response options of “Yes,” “No,” or “I do not know.” The third question was, “What is the cause of a PEIR lesion?” The answer options were “Localized developmental anomaly,”, “Infective primary tooth,” “Caries,” “Fluoride exposure,” “Resorption,” and “I do not know.” The fourth question was, “Do you think it is important for dental practitioners to know about PEIR?” with response options of “Yes” or “No.”

Radiographs for Cases #2 and #4 were obtained from the institutional archives. The remaining radiographs were reproduced from published sources with permission obtained from the corresponding authors, and each source is cited below the relevant radiograph. The radiographic assessment was conducted under standardized viewing conditions in a fixed, dimly lit office with no distractions within the observers' field of view. Radiographs were displayed on a 15.6-inch laptop screen (Latitude E6530, Dell Corporation, Round Rock, TX, USA) with a resolution of 1,600 × 900 pixels. The laptop screen was positioned perpendicular to the floor, and the viewing distance between the participant's eyes and the screen was adjusted to 50–75 cm. Participants completed the assessment independently without discussion and were instructed to examine the radiographs as they would in clinical practice. No time limit was imposed for the assessment of each radiograph.

### 2.6 Statistical analysis

The data were analyzed using the Statistical Package for the Social Sciences (SPSS®; IBM, Endicott, NY, USA), version 25. Descriptive statistics were calculated and presented as frequencies and percentages. Associations between participants' overall scores for detection/identification, diagnosis, severity assessment, and management were evaluated according to educational level and years of clinical experience using the nonparametric Kruskal–Wallis test, followed by *post hoc* analyses when applicable. The significance level was set at *p* < 0.05.

## 3 Results

### 3.1 Study population

A total of 163 dentists and dental interns were invited to participate in this study, of whom 125 completed the survey, yielding a response rate of 76.7%. The demographic characteristics and clinical experience of the participants are summarized in Table 1. Females comprised 60.0% of the study population, while males accounted for 40.0%. Half of the participants (50.4%) were between 26 and 30 years of age. Based on educational level, participants were classified as dental interns (37.6%), general dentists (20.8%), pediatric dentistry residents (19.2%), and residents from other dental specialties (22.4%). Nearly all participants (99.2%) obtained their undergraduate dental degree in Saudi Arabia, and clinical experience ranged from one year (37.6%) to ≥5 years (30.4%).

**Table 1:** Demographic characteristics and clinical experience of the study participants.

Variable	Categories	Total
Gender	Male	50 (40.0%)
	Female	75 (60.0%)
Age	20–25 years	44 (35.2%)
	26–30 years	63 (50.4%)
	≥ 31 years	18 (14.4%)
Level of education	Interns	47 (37.6%)
	General dentists	26 (20.8%)
	Pediatric residents	24 (19.2%)
	Other residents	28 (22.4%)
Undergraduate education	KSA	124 (99.2%)
	Other	1 (0.8%)
Clinical experience	1 year	47 (37.6%)
	2–4 years	40 (32.0%)
	≥ 5 years	38 (30.4%)

### 3.2 Radiographic detection and diagnostic performance

The distribution of correct responses for radiographic detection, diagnosis, PEIR severity assessment, and management is presented in Table 2.

**Table 2:** Correct responses to radiographic detection, diagnosis, PEIR severity assessment, and management across the study scenarios.

Scenario	Case	Description	Correct Detection/Identification n (%)	Correct Diagnosis (Out of detected)/Identified	Correct Severity Assessment (out of Identified)	Correct Management Decision (out of Identified)
1	Case 1	Normal radiograph	90 (72%)	—	—	—
	Case 2	Impacted tooth #35	123 (98.4%)	122/123 (99.2%)	—	—
	Case 3	Moderate PEIR	11 (8.8%)	11/11 (100%)	—	—
2	Case 4	Odontodysplsia	107 (85.6%)	104/107 (97.2%)	—	—
	Case 5	Moderate PEIR	21 (16.8%)	—	—	—
3	Case 6	Mild PEIR	79 (63.2%)	48/79 (60.8%)	61/79 (77.2%)	—
	Case 7	Moderate PEIR	68 (54.4%)	49/68 (72.1%)	40/68 (58.8%)	—
	Case 8	Severe PEIR	106 (84.8%)	82/106 (77.4%)	34/106 (32.1%)	—

Participants showed high accuracy in recognizing normal radiographic findings and impacted teeth. Approximately three-quarters (72.0%) correctly identified the normal panoramic radiograph in Case 1, whereas nearly all participants (98.4%) correctly detected the impacted tooth in Case 2. Diagnostic accuracy was also high for odontodysplasia (85.6% in Case 4). In contrast, identification of moderate PEIR lesions was poor in the initial scenarios, with only 8.8% and 16.8% of participants correctly identifying the affected tooth in Cases 3 and 5, respectively.

Among participants who correctly detected the abnormalities, diagnostic accuracy was excellent. Of the 123 participants who correctly detected the impacted tooth in Case 2, only one participant provided an incorrect diagnosis. Likewise, all participants who correctly identified the PEIR lesion in Case 3 correctly diagnosed the condition, while only three participants misdiagnosed the odontodysplasia case after correctly detecting the abnormality.

When participants were informed that the radiographs contained PEIR lesions (Cases 6–8), detection rates improved, with correct identification rates of 63.2% for mild lesions, 54.4% for moderate lesions, and 84.8% for severe lesions. Among those who correctly identified the affected teeth, the accuracy of severity assessment ranged from 60.8% to 77.4%. Management decisions varied according to lesion severity. Follow-up until eruption was correctly selected by 77.2% of participants for mild lesions and 58.8% for moderate lesions, whereas only 32.1% correctly selected surgical intervention and restoration before eruption for severe PEIR lesions.

### 3.3 Overall radiographic diagnostic performance

The overall radiographic diagnostic performance of the participants is summarized in Table 3. No statistically significant differences were observed in the radiographic detection score according to educational level or clinical experience. However, the diagnosis score differed significantly according to educational level (*p* = 0.015), with pediatric dentistry residents achieving a significantly higher mean score than dental interns (2.13 ± 0.45 vs. 1.72 ± 0.54).

**Table 3:** Overall radiographic diagnostic performance according to educational level and clinical experience.

Variable	Detection Score/8	Diagnosis Score/3	Total Score/11
Overall	4.84 ± 1.47	1.90 ± 0.54	6.74 ± 1.84
Level of education			
Interns	4.49 ± 1.33	1.72 ± 0.54^a^	6.21 ± 1.64
General dentists	5.00 ± 1.36	1.92 ± 0.48^ab^	6.92 ± 1.65
Pediatric residents	5.08 ± 1.59	2.13 ± 0.45^b^	7.21 ± 1.93
Other residents	5.07 ± 1.63	1.96 ± 0.58^ab^	7.04 ± 2.10
*P*-value§	0.178	0.015*	0.070
Clinical experience			
1 y	4.49 ± 1.33	1.72 ± 0.54^a^	6.21 ± 1.64^a^
2–4 y	5.03 ± 1.31	1.90 ± 0.44^ab^	6.93 ± 1.59^ab^
≥ 5 y	5.08 ± 1.72	2.11 ± 0.56^b^	7.18 ± 2.17^b^
*P*-value§	0.085	0.004*	0.027*

Values are presented as mean ± standard deviation. *P* values were calculated using the Kruskal–Wallis test. Different superscript letters indicate statistically significant differences between groups according to the *post hoc* analysis.

Clinical experience was also associated with diagnostic performance. Participants with ≥5 years of clinical experience achieved significantly higher diagnosis scores than those with 1 year of experience (2.11 ± 0.56 vs. 1.72 ± 0.54; *p* = 0.004). Similarly, the overall radiographic diagnostic score was significantly higher among participants with ≥5 years of clinical experience than among those with 1 year of experience (7.18 ± 2.17 vs. 6.21 ± 1.64; *p* = 0.027).

### 3.4 PEIR-related diagnostic performance

The PEIR-related diagnostic performance of the participants is summarized in Table 4. No statistically significant differences were observed in the PEIR identification or severity assessment scores according to educational level or clinical experience.

**Table 4:** PEIR-related diagnostic performance according to educational level and clinical experience.

Variable	PEIR Identification Score(/5)	PEIR Severity Score (/3)	PEIR Management Score (/3)	PEIR Total Score (/11)
Overall	2.28 ± 1.23	2.35 ± 0.84	1.73 ± 0.99	7.54 ± 1.47
Level of education				
Interns	2.11 ± 1.13	2.20 ± 1.01	1.33 ± 1.11^a^	6.93 ± 1.71^a^
General dentists	2.38 ± 1.24	2.33 ± 0.78	1.83 ± 0.72^ab^	7.58 ± 0.99^ab^
Pediatric Residents	2.25 ± 1.42	2.50 ± 0.85	2.60 ± 0.52^b^	8.80 ± 1.23^b^
Other Residents	2.50 ± 1.23	2.40 ± 0.74	1.47 ± 0.99^a^	7.23 ± 1.28^ab^
*P*-value§	0.532	0.840	0.006*	0.027*
Clinical experience				
1 y	2.11 ± 1.13	2.20 ± 1.01	1.33 ± 1.11^a^	6.93 ± 1.71^a^
2–4 y	2.33 ± 1.12	2.25 ± 0.72	1.65 ± 0.93^ab^	7.15 ± 1.04^a^
≥ 5 y	2.45 ± 1.45	2.59 ± 0.80	2.18 ± 0.81^b^	8.53 ± 1.23^b^
*P*-value §	0.491	0.194	0.047*	0.004*

Values are presented as mean ± standard deviation. *P* values were calculated using the Kruskal–Wallis test. Different superscript letters indicate statistically significant differences between groups according to the *post hoc* analysis.

However, the PEIR management score differed significantly according to educational level (*p* = 0.006). Pediatric dentistry residents achieved significantly higher mean management scores than dental interns (2.60 ± 0.52 vs. 1.33 ± 1.11) and residents from other dental specialties (2.60 ± 0.52 vs. 1.47 ± 0.99). Clinical experience was also associated with PEIR management performance, with participants having ≥5 years of clinical experience achieving significantly higher management scores than those with 1 year of experience (2.18 ± 0.81 vs. 1.33 ± 1.11; *p* = 0.047).

Similarly, the overall PEIR score differed significantly according to educational level and clinical experience. Pediatric dentistry residents achieved significantly higher total PEIR scores than dental interns (8.80 ± 1.23 vs. 6.93 ± 1.71; *p* = 0.027), while participants with ≥5 years of clinical experience had significantly higher total PEIR scores than those with fewer years of experience (*p* = 0.004).

### 3.5 General knowledge about PEIR

The participants' responses to the general questions regarding PEIR are summarized in Table 5. Only 24.8% of participants reported having previously heard of PEIR, and just 6.4% indicated that they had encountered a PEIR lesion in clinical practice, while 74.4% reported no previous clinical experience with the condition. Among those who had encountered PEIR clinically, six were pediatric dentistry residents and two were dental interns. Only 8.8% of participants correctly identified resorption as the etiology of PEIR. Nevertheless, almost all participants (98.4%) acknowledged the importance of dental practitioners being knowledgeable about PEIR.

**Table 5:** General knowledge and clinical experience regarding PEIR.

Question	Response	*n*	%
Q1. Have you ever heard of Pre-eruptive Intra-coronal Resorption (PEIR)?	Yes	31	24.8
	No	94	75.2
Q2. Have you ever encountered a PEIR lesion in your clinic?	Yes	8	6.4
	No	93	74.4
	I don't know	24	19.2
Q3. What is the cause of a PEIR lesion?	Correct (Resorption)	11	8.8
	Incorrect answers	114	91.2
Q4. Do you think it is important for dental practitioners to know about PEIR?	Yes	123	98.4
	No	2	1.6

Values are presented as frequencies (n) and percentages (%).

## 4 Discussion

The objective of this study was to assess the ability of dental practitioners at KAUDH to detect and diagnose PEIR and to evaluate their knowledge of PEIR etiology, severity, and management. The influence of factors including educational level and years of clinical experience on diagnostic performance and the accuracy of radiographic interpretation was also assessed. The results showed that the participants had limited knowledge of PEIR. The findings revealed an association between PEIR lesion severity and the detection of PEIR-affected teeth; as lesion severity increased, participants were better able to identify the affected teeth. However, as lesion severity increased, participants' awareness regarding the appropriate management decreased. The results also showed that educational level and years of clinical experience were among the factors affecting diagnostic accuracy and radiographic interpretation.

To our knowledge, only one published study has assessed practitioners' knowledge of PEIR in South India using a self-constructed 15-item closed-ended questionnaire (16). In the present study, a validated questionnaire was used. Face validity, content validity, and test–retest reliability of the questionnaire were evaluated. The questionnaire was developed following a thorough review of the literature. It consisted of three different scenarios with varying levels of case difficulty to assess practitioners' knowledge of PEIR.

The first scenario consisted of four cases (Case #1: normal radiograph, Case #2: impacted tooth, Case #3: moderate PEIR lesion, and Case #4: odontodysplasia). The purpose of this scenario was to compare the participants' ability to detect and diagnose normal and abnormal radiographs. It also aimed to compare their ability to detect and diagnose obvious abnormalities (Cases #2 and #4) with their ability to identify the PEIR lesion in Case #3, in which the abnormality was less apparent. The results for the normal radiograph (Case #1) showed that 72.0% of the participants correctly identified the absence of abnormalities on the panoramic radiograph. The results for the two cases with obvious abnormalities (Cases #2 and #4) showed high rates of correct detection and diagnosis. The percentages of correct detection in Cases #2 and #4 were 98.4% and 85.6%, respectively, whereas the percentages of correct diagnosis were 99.2% and 97.2%, respectively. These findings indicate that the participants demonstrated a high level of proficiency in the diagnosis and interpretation of common dental abnormalities. Although the PEIR lesion in Case #3 was moderate in severity, only 8.8% of the participants were able to identify the affected tooth. This finding may be attributed to the subtle radiographic appearance of the lesion, which made its recognition and identification more challenging. These findings suggest that participants experienced difficulty recognizing the subtle radiographic features of PEIR, particularly when the lesion was not explicitly highlighted, while demonstrating adequate recognition of more obvious abnormalities.

The purpose of the second scenario (Case #5, moderate PEIR lesion) was to evaluate the effect of informing participants that the radiograph contained an abnormality on their ability to identify the tooth affected by PEIR. Although the participants were informed that the radiograph contained an abnormality and were asked to identify the affected tooth, only 16.8% were able to do so. These findings were similar to the results obtained from Case #3 and confirm that the knowledge of PEIR was limited among the participants.

The purpose of the third scenario (Cases #6–8, arranged according to PEIR severity) was to assess the ability of dental practitioners to identify teeth affected by PEIR, as well as to evaluate their knowledge of PEIR severity and management. The results showed that, as PEIR severity increased, participants were more likely to identify the affected teeth (63.2% in Case #6 and 84.8% in Case #8). These findings may be explained by the fact that larger PEIR lesions produce more extensive radiolucency and greater loss of dentinal structure, making them more conspicuous on panoramic radiographs. In contrast, mild lesions are often subtle and may be overlooked during routine radiographic assessment. Similar findings have been reported in studies evaluating radiographic interpretation, in which diagnostic performance improved when abnormalities were more visually apparent and declined with increasing case complexity (18). However, Case #7 did not follow this trend, as only 54.4% of the participants were able to identify the affected tooth. A possible explanation for this finding is that Case #7 contained a greater number of unerupted teeth than Case #6. Moreover, as the severity of Cases #6–8 increased, the percentages of correct answers to the severity assessment question also increased (60.8%, 72.1%, and 77.4%, respectively). However, the percentages of correct answers regarding the management of Cases #6–8 decreased (77.2%, 58.8%, and 32.1%, respectively). These findings suggest that although participants were increasingly able to recognize more severe PEIR lesions radiographically, selecting the appropriate management strategy became more challenging as lesion severity increased. Management of advanced PEIR lesions requires a comprehensive understanding of lesion progression, eruption timing, the risk of pulpal involvement, and the indications for surgical intervention versus monitoring. Consequently, treatment planning may require a higher level of clinical knowledge and decision-making skills than lesion detection alone, which could explain the decline in management accuracy observed in the more severe cases. Similarly, Bahaziq et al. reported that diagnostic performance during panoramic radiograph interpretation varied according to case complexity, with more conspicuous findings being identified more readily than subtle abnormalities (18).

In this study, we included different cases of PEIR with different degrees of severity and management requirements. No previous studies with a similar design were identified for comparison with our findings regarding PEIR detection, severity assessment, and management. However, Sagar and Deepa investigated practitioners' knowledge of PEIR management in general and reported that approximately 41% of participants considered extraction to be the treatment of choice, followed by restoration (36%) (16).

In terms of diagnosis, the findings revealed that pediatric residents had a significantly higher mean diagnostic score compared to interns. In addition, participants with ≥5 years of clinical experience had significantly higher mean diagnostic and total scores than participants with 1 year of clinical experience. Furthermore, regarding the diagnostic performance, pediatric residents had a significantly higher mean management score than residents from other specialties and interns. Similarly, the mean management score of participants with ≥5 years of clinical experience was significantly higher than that of participants with 1 year of clinical experience. Pediatric residents also had a significantly higher mean PEIR total score than interns. In addition, participants with ≥5 years of clinical experience had significantly higher mean PEIR total scores than the other experience groups. Similarly, Turgeon and Lam found that the performance of oral and maxillofacial radiology residents was better than that of students, which they attributed to differences in knowledge and training (19). In contrast to our findings, Bahaziq et al. found no significant differences among the groups in the recognition of incidental findings with varying levels of difficulty (18). This discrepancy may be explained by the comparable levels of knowledge and radiographic interpretation skills among the groups evaluated in their study.

In the present study, only 24.8% of the participants had heard of PEIR, and 74.4% reported never encountering a PEIR lesion in their clinical practice. These findings indicate limited awareness of and clinical exposure to PEIR among the surveyed dentists. In contrast, Sagar and Deepa reported higher awareness levels, with 61% of participants having heard of PEIR and 23% reporting recognition of the condition through clinical experience (16). The higher awareness reported in the South Indian study may be attributed to regional differences in dental educational systems and undergraduate training exposure, where rare developmental conditions such as PEIR may receive varying levels of emphasis within pediatric dentistry or oral pathology curricula. Furthermore, direct comparison should be made cautiously, as their study did not account for participants' demographic characteristics, educational level, or years of clinical experience. Unmeasured differences in sample composition, such as a greater proportion of specialists, postgraduate trainees, or practitioners working in academic hospital settings, may have contributed to the higher levels of clinical exposure observed in their study compared with the present study population. Regarding the perceived etiology of PEIR, 44% of participants in the current study were uncertain, 32% considered it a localized developmental anomaly, and only 8.8% correctly identified resorption as the underlying mechanism. Similar trends were observed by Sagar and Deepa, who reported that 41% of respondents attributed PEIR to developmental defects, whereas approximately 20% recognized resorption as its cause. The limited recognition of PEIR observed in this study has important clinical implications, as delayed diagnosis may allow lesion progression, thereby increasing the risk of pulpal involvement and necessitating more complex treatment. The findings also suggest that undergraduate dental training may provide limited exposure to uncommon developmental anomalies such as PEIR, which could contribute to reduced awareness and diagnostic confidence. To address these challenges, greater emphasis on case-based learning, radiographic interpretation training, and continuing professional education may improve practitioners' diagnostic competence and facilitate the earlier detection and appropriate management of PEIR lesions.

The present study has several limitations that should be acknowledged. First, the single-center design limits the generalizability of the findings to the broader population of dental practitioners across Saudi Arabia. However, an effort was made to mitigate this limitation by recruiting a diverse group of participants with varying educational backgrounds and levels of clinical experience. An additional limitation is the exclusive use of panoramic radiographs for case evaluation. Because panoramic imaging provides only a two-dimensional representation, diagnostic accuracy may have been reduced. The use of cone-beam computed tomography in future studies may provide greater diagnostic sensitivity and three-dimensional accuracy for the detection of PEIR lesions. Furthermore, because participation was voluntary, practitioners with greater interest in radiographic interpretation or greater confidence in identifying abnormalities may have been more likely to participate. This may have introduced self-selection bias and led to an overestimation of knowledge and diagnostic performance. Another limitation relates to the sample size available for subgroup analyses. Although the sample size calculation was performed *a priori* and was based on detecting differences in overall questionnaire scores between the main groups, several analyses were conducted after stratification by educational level and years of clinical experience. Some of these subgroups contained relatively small numbers of participants, which may have reduced statistical power and increased uncertainty around the estimated differences. Therefore, findings related to subgroup comparisons should be interpreted cautiously and considered exploratory. Another methodological limitation relates to the questionnaire itself. Although the questionnaire underwent an initial validation process, including face validity assessment, expert-based content validity assessment, and test–retest reliability evaluation, it was not subjected to comprehensive psychometric validation. Despite these limitations, the study has several strengths, including a high response rate, with 76.69% of invited dentists participating. Overall, this study highlights the importance of incorporating knowledge of rare conditions such as PEIR into dental curricula. Increasing awareness may facilitate earlier detection and appropriate management, thereby improving prognostic outcomes. These findings may also be valuable to healthcare authorities seeking to promote evidence-based dentistry among practitioners in Saudi Arabia.

## 5 Conclusions

This study identified important gaps in awareness and conceptual understanding of PEIR among dental practitioners, particularly regarding its etiology and management. Although recognition of subtle PEIR lesions was limited, participants demonstrated improved detection as lesion severity increased, suggesting that radiographic identification is influenced by lesion presentation rather than solely by overall knowledge of the condition. More experienced clinicians and pediatric dentistry residents appeared to perform comparatively better in some analyses, suggesting a possible role for clinical exposure and specialized training; however, these subgroup findings should be interpreted cautiously because of the limited sample size in some strata. The subtle radiographic appearance of PEIR makes its detection challenging, which may contribute to delayed diagnosis and management. These findings emphasize the need to strengthen educational and clinical training programs to improve practitioners'' ability to identify and appropriately manage PEIR lesions at an early stage. These findings support the incorporation of PEIR and other uncommon developmental dental anomalies into undergraduate curricula, as well as the provision of continuing professional education focused on radiographic interpretation and early diagnosis.

## Data Availability

The raw data supporting the conclusions of this article will be made available by the authors, without undue reservation.

## References

[1] Almashaikhi J Elkhodary HM Bhadila GY Felemban OM Al Tuwirqi AA. Diagnostic accuracy and radiographic interpretation of pre-eruptive intra-coronal resorption among dental practitioners using eye-tracking technology. Digit Health. (2025) 11:20552076251315620. 10.1177/20552076251315620 39949845 PMC11822827

[2] Al-Batayneh O AlTawashi E. Pre-eruptive intra-coronal resorption of dentine: a review of aetiology, diagnosis, and management. Eur Arch Paediatr Dent. (2020) 21(1):1–11. 10.1007/s40368-019-00470-4 31414395

[3] Al-Tuwirqi A Seow WK. A controlled study of Pre-eruptive intracoronal resorption and dental development. J Clin Pediatr Dent. (2017) 41(5):374–380. 10.17796/1053-4628-41.5.374 28872985

[4] Moura BF Silveira GR Rodrigues-Junior SA. Diagnosis and clinical management of pre-eruptive intracoronal resorption–a case report. Rev Sul-Bras Odontol. (2016) 13(2):109–15. 10.21726/rsbo.v13i2.280

[5] Gera A Zilberman U. Diagnosis and management of hidden caries in a primary molar tooth. Int J Clin Pediatr Dent. (2017) 10(1):99. 10.5005/jp-journals-10005-1415 28377664 PMC5360812

[6] Al-Batayneh O AlJamal G AlTawashi E. Pre-eruptive intracoronal dentine radiolucencies in the permanent dentition of Jordanian children. Eur Arch Paediatr Dent. (2014) 15(4):229–36. 10.1007/s40368-013-0104-x 24353075

[7] Uzun İ Gunduz K Canitezer G Avsever H Orhan K. A retrospective analysis of prevalence and characteristics of pre-eruptive intracoronal resorption in unerupted teeth of the permanent dentition: a multicentre study. Int Endod J. (2015) 48(11):1069–76. 10.1111/iej.12404 25354246

[8] Demirtas O Dane A Yildirim E. A comparison of the use of cone-beam computed tomography and panoramic radiography in the assessment of pre-eruptive intracoronal resorption. Acta Odontol Scand. (2016) 74(8):636–41. 10.1080/00016357.2016.1235227 27669814

[9] Umansky M Tickotsky N Friedlander-Barenboim S Faibis S Moskovitz M. Age related prevalence of pre-eruptive intracoronal radiolucent defects in the permanent dentition. J Clin Pediatr Dent. (2016) 40(2):103–6. 10.17796/1053-4628-40.2.103 26950809

[10] Manmontri C Chompu-inwai P Mahasantipiya PM Prapayasatok S. Prevalence of pre-eruptive intracoronal radiolucencies in Thai children and adolescents: a retrospective study. J Investig Clin Dent. (2018) 9(2):e12303. 10.1111/jicd.12303 29055115

[11] Konde S Sri Darshini CS Agarwal M Peethambar P. Unrevealed caries in unerupted teeth: a prevalence study. Contemp Clin Dent. (2018) 9(Suppl 2):S305–8. 10.4103/ccd.ccd_291_18 30294162 PMC6169283

[12] Seow WK Wan A McAllan LH. The prevalence of pre-eruptive dentin radiolucencies in the permanent dentition. Pediatr Dent. (1999) 21(1):26–33. 10029964

[13] Özden B Acikgoz A. Prevalence and characteristics of intracoronal resorption in unerupted teeth in the permanent dentition: a retrospective study. Oral Radiol. (2009) 25(1):6–13. 10.1007/s11282-009-0003-3

[14] Kjær I Steiniche K Kortegaard U Pallisgaard C Bille M-LB Seirup T. Preeruptive intracoronal resorption observed in 13 patients. Am J Orthod Dentofacial Orthop. (2012) 142(1):129–32. 10.1016/j.ajodo.2011.03.026 22748998

[15] Barra SG Villalobos MIOB Penido CVSR Recchioni ACB Visconti MAPG. Pre-interruptive intracoronal resorption: clinical and radiographic follow-up. Rev Gaúch Odontol. (2017) 65(03):282–5. 10.1590/1981-863720170002000163430

[16] Sagar S Deepa G. Knowledge of pre-eruptive intracoronal resorption among dental practitioners–A questionnaire-based study. Drug Invent Today. (2020) 14(3):340–4.

[17] Emmanuel A Clow SE. A questionnaire for assessing breastfeeding intentions and practices in Nigeria: validity, reliability and translation. BMC Pregnancy Childbirth. (2017) 17(1):174. 10.1186/s12884-017-1366-9 28592252 PMC5463374

[18] Bahaziq A Jadu FM Jan AM Baghdady M Feteih RM. A comparative study of the examination pattern of panoramic radiographs using eye-tracking software. J Contemp Dent Pract. (2019) 20(12):1436–41. 10.5005/jp-journals-10024-2700 32381846

[19] Turgeon DP Lam EW. Influence of experience and training on dental students' Examination performance regarding panoramic images. J Dent Educ. (2016) 80(2):156–64. 10.1002/j.0022-0337.2016.80.2.tb06071.x 26834133

